# Temporal binding and sense of agency in major depression

**DOI:** 10.3389/fpsyt.2024.1288674

**Published:** 2024-04-05

**Authors:** David H. V. Vogel, Mathis Jording, Peter H. Weiss, Kai Vogeley

**Affiliations:** ^1^ Research Center Jülich, Institute of Neuroscience and Medicine, Cognitive Neuroscience (INM-3), Juelich, Germany; ^2^ Department of Psychiatry, Faculty of Medicine and University Hospital Cologne, University of Cologne, Cologne, Germany; ^3^ Department of Neurology, Faculty of Medicine and University Hospital Cologne, University of Cologne, Cologne, Germany

**Keywords:** sense of agency (SoA), self-efficacy (SE), temporal binding (TB), time perception, major depressive disorder (MDD)

## Abstract

**Background:**

Alterations in the experience of controlling oneself and one’s environment are of high relevance to understanding the psychopathology of depression. This study investigated the relationship between Temporal Binding for action-event sequences, sense of agency, self-efficacy and symptom severity in Major Depressive Disorder.

**Method:**

We employed the Sense of Agency Scale (SoAS) and the General Self-Efficacy Scale (GSE) to assess explicit Sense of Agency and self-efficacy in a group of 42 persons diagnosed with Major Depressive Disorder (MDD) [20 identifying as female, 19 as male; mean age 37.8 years (± 13.3)] and 40 control persons without a psychiatric diagnosis (CG) [22 identifying as female, 20 as male; mean age 38.0 years ( ± 13.3)]. Depressive symptom severity was measured using the BDI-II. We additionally performed a temporal binding paradigm as a potential correlate to Sense of Agency. Participants partook in a time estimation task judging three intervals (250ms, 450ms, 650ms) while either observing or causing stimulus presentations. The underestimation of intervals following intentional actions causing stimulus presentations (compared to merely observing the stimulus presentation) is interpreted as temporal binding.

**Results:**

SoAS scores demonstrated an inverse correlation with depressive symptoms (CG: p=.032, R^2^=.113; MDD: p<.001, R^2^=.260) and a positive correlation with GSE scores (CG: p<.001, R^2^=.379; MDD: p<.001, R^2^=.254). We found distinct differences in temporal binding between healthy participants and the Major Depressive Disorder group without significant correlation between temporal binding and the SoAS or GSE scores. The data suggest group differences in time estimation particular pertaining to time intervals involving intentional action and increasingly complex multisensory stimuli.

**Discussion:**

We investigated parameters of subjective control, namely Sense of Agency and Self Efficacy. Here, we were able to reveal their inverse relationship with depressive symptoms in patients with major depressive disorder, highlighting a profound experience of loss of control with increasing symptom load. Deficits in experiencing control, particularly involving intentional motor actions (and more complex multisensory stimuli), appear to be more pronounced in Major Depressive Disorder, involving not only negative self-efficacy expectations but also an altered Sense of Agency and temporal binding. Temporal binding and SoAS scores did not correlate, adding to the growing evidence that the two measures may not be directly related. We propose that future research be directed at this contiguous relationship between Sense of Agency and Self Efficacy in Major Depressive Disorder.

## 1 Introduction

The experience of self-efficacy (SE), as the expectation of one’s own actions to be successful, is commonly reported to be negatively affected in major depressive disorder (MDD) (1). This decrease holds diagnostic and therapeutic relevance due to its relation to decreased motivational factors (2, 3). The definition of self-efficacy is closely related to the Sense of Agency (SoA), which most commonly refers to being the person or entity intending and performing an action and hence feeling responsible for its outcome (4).

While both self-efficacy and Sense of Agency are linked to an experience of having control over the external world through one’s own behavior, and are psychometrically related (5) both concepts describe distinct phenomena (6). While self-efficacy refers to the expected control over being able to reach a desired goal or meaningful outcome, Sense of Agency entails the feeling of control over any type of intentional action and its expected result. Accordingly, self-efficacy experiences refer to the felt capacity to reach a goal prior to and during a performed action, while Sense of Agency refers to experienced control during ongoing goal directed motor actions and afterwards (4). As in both cases perceived control and outcome prediction are essential, both concepts are often used almost interchangeably, depending on context and usage (4, 7). In such instances, the distinction between Sense of Agency and self-efficacy may appear subtle (8, 9, p. xv). Conversely, this usage also indicates conceptual and potentially procedural overlap between Sense of Agency and self-efficacy (10).

With respect to clinical observations, it is interesting to note, that self-efficacy (rather than Sense of Agency) is reported to be altered in major depressive disorder, while Sense of Agency (rather than self-efficacy) is more commonly altered in schizophrenia (11–15). But, for both self-efficacy and Sense of Agency, the consequence of their reduction is a patient’s experience of losing control over their physical environment and social context. This observation has led cognitive scientists to expect Sense of Agency alterations in major depressive disorder (16–18). A particularly interesting question concerns the relationship between self-efficacy and Sense of Agency under the assumption of a common mechanism leading to the experience of loss of control in major depressive disorder (10). Current literature differentiates between two distinct ways of assessing Sense of Agency. *Explicit* Sense of Agency refers to judgements on experienced control and authorship, usually quantified with visual analogue scales – as a state dependent variable – or specific questionnaires – measuring trait dependence. *Implicit* Sense of Agency is assessed with the temporal binding (TB) effect, originally referred to as “intentional binding” (19). It is often measured by means of time estimation tasks (20, 21) and describes the underestimation of time intervals between voluntary actions and their effects (e.g., time interval between a button press and a subsequent tone), as compared to estimates of durations between two events occurring independent from one´s own voluntary action (e.g., time interval between two tones).

Importantly, temporal binding is not an uncontested, direct correlate to Sense of Agency. It may also occur in contexts not involving action-event succession. Temporal binding more likely corresponds to causation, and the underlying involvement of neural multisensory processes (e.g., 22–27). The effect comes about whenever two successive events stand in a causal relationship. Under the assumption that e.g., a predictable event following an intentional action is likely to happen, thus causally related events are processed in unison, even across a short time interval — the temporal binding window (28). With more predictable event sequences temporal binding will be stronger (27, 29, 30).

Action-event sequences constitute a subset of such causal orders involving separate mechanisms (31). Accordingly, temporal binding and implicit Sense of Agency are not entirely synonymous (23, 24, 32). Intentional action involves more available information, involving both prior top-down information such as motor and outcome planning, and bottom-up perceptual information such as proprioception, visual and sensory information. With more information, predictability of the event sequence increases (33) and with more information being involved in self-performed action stronger temporal binding emerges (22, 25, 34, 35). In other words, temporal binding provides information on how information is processed during causal action event sequences, including those involving intentional action.

Despite this rich theory there is currently no empirical knowledge available concerning the relationship between temporal binding, Sense of Agency, and self-efficacy in patients with major depressive disorder. By combining established Sense of Agency and self-efficacy measures it may be possible to identify hitherto undetected behavioral and experiential correlates of depressive symptoms.

We propose that self-efficacy and Sense of Agency are closely related, since self-efficacy and Sense of Agency - at their core - relate to a similar experience affected by major depressive disorder, namely the loss of control. Thus, here we combine self-efficacy, Sense of Agency and temporal binding measures in patients with major depressive disorder to further explore their (potential) experience of loss of control employing both explicit, experiential methods and implicit, behavioral measures.

## 2 Materials and methods

We took measures of 4 different variables from a group of patients diagnosed with major depressive disorder and a group of healthy non-depressed individuals. We applied three self-reporting questionnaires: the BDI-II for depressive symptoms and symptom severity (36), the Sense of Agency Scale (SoAS) for subjective agency ratings (37), and the General Self-Efficacy Scale (GSE) for subjective self-efficacy ratings (38, 39). Additionally, we conducted a time estimation experiment as a measure of temporal binding.

### 2.1 Questionnaires

The BDI-II (36) consists of 21 questions with four answers to be scored from 0 to 3. Questions address depressive symptoms; the score represents symptom severity. Higher scores indicate more severe depressive symptoms.

The SoAS is a recently developed scale addressing Sense of Agency (37). It is specifically designed to measure subjective “core” Sense of Agency independent from “instrumentality or goal-directedness” with higher scores indicating stronger core Sense of Agency. The scale can be separated into a negative Sense of Agency scale and a positive Sense of Agency scale. In non-depressed subjects the SoAS does not systematically correlate with depressive symptoms (37). To our knowledge, the SoAS has not been applied to patients with major depressive disorder.

The GSE is a frequently used and validated scale to assess self-efficacy (38). Higher scores indicate high subjective self-efficacy, while lower scores indicate lower subjective self-efficacy. GSE scores have been shown to correlate with depressive symptoms (40).

For BDI-II and GSE the validated German versions were used. The SoAS was translated and verified by back-translation but has not been validated for application in a German speaking population.

### 2.2 Experimental procedure

The experimental paradigm used in this study was based on previous studies investigating temporal binding with both visual and auditory stimuli (41, 42). As this study, to our knowledge, is the first study investigating temporal binding in patients with major depressive disorder, we decided to use a comprehensive paradigm involving three factors, i.e., (a) AGENCY (operant vs. observant), (b) STIMULUS (visual vs. auditory), and (c) INTERVAL (250ms, 450ms, 650ms) across two DIAGNOSES (CG vs. MDD). We adopted this comparatively complex study design to ensure that we accounted for potential alterations in implicit Sense of Agency across different stimulus modalities and across time intervals. We selected three intervals to cover a broader range of potentially affected durations and selected two stimulus modalities to account for potential differences in multisensory processing by covering more than one sensory input modality. Thereby, we hoped to increase the sensitivity to detect altered temporal binding in major depressive disorder, as temporal binding paradigms have not been applied in this patient group before.

Participants were seated in front of a computer screen and instructed to repeatedly estimate the duration between two events. In the 2 x 2 x 3 factorial design, the factor AGENCY was implemented by varying the first event of each sequence. This initial event was either a 1 kHz tone played over headphones for 100ms, between 1.5 to 2 seconds after trial start, or a button press (space bar on a computer keyboard) performed voluntarily without instruction by the participant at a time of their choosing. Conditions were labelled *observant* (tone without button press) and *operant* (button press; factor AGENCY). The first event was then followed by an empty time interval of either 250ms, 450ms, or 650ms (factor INTERVAL). The second event consisted of either a *visual* stimulus (a red dot with a diameter of approximately 2.5 degrees visual angle flashing once for 100ms at the center of the screen), or an *auditory* stimulus (a 1.5 kHz beep audible for 100ms; factor STIMULUS). AGENCY conditions were presented separately in two blocks, while the factors STIMULUS and INTERVAL were presented in randomized order within blocks. The two blocks [AGENCY (operant vs. observant)] were counterbalanced across subjects and consisted of 60 trials resulting in 10 trials per separate condition [i.e., STIMULUS (visual vs. auditory), and INTERVAL (250ms, 450ms, 650ms)]. Each block lasted a maximum of 15 minutes resulting in a maximum duration of the experiment of 30 minutes.

**Figure 1:**
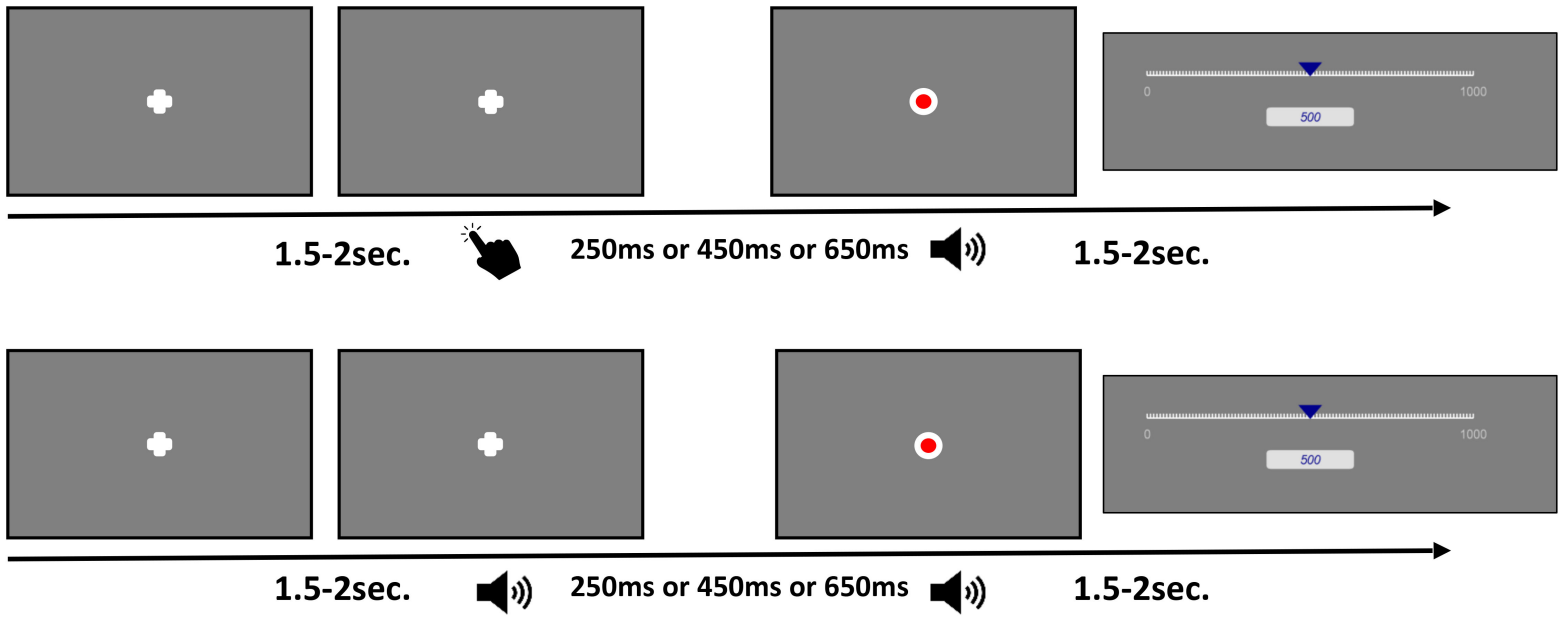
Trial event structure. The *top row* depicts trials for the operant, active block. After a voluntary period, participants pressed a key resulting in either a tone or a flash on the screen after one of three intervals. Afterwards participants were instructed to estimate the time between key press and the subsequent stimulus presentation using a visual analogue scale. The *bottom row* depicts trials for the observant, passive block. After 1.5 - 2 seconds a tone was played resulting in either a tone or a flash on the screen after one of three intervals. Afterwards participants were instructed to estimate the time between the initial tone and the subsequent stimulus presentation using a visual analogue scale.

After completion of each trial, a visual analogue scale appeared after a random duration between 1.5 to 2.5 seconds. The bottom anchor of the visual analogue scale was 0ms representing the perception of immediacy. The top anchor was 1000ms. We reminded participants that 1 second contained 1000ms and that the visual analogue scale represented one second. Participants clicked on the visual analogue scale using their computer mouse after which a blue indicator appeared on the scale. A numerical estimate simultaneously appeared below the scale corresponding to the indicators position on the scale. Estimate could then be adjusted by further clicking on the scale and moving the cursor. Participants were instructed to estimate the time interval between the initial tone (for *observant* trials) or their key press (for *operant* trials) and the subsequent event (dot or tone) in milliseconds on the visual analogue scale. Participants did not undergo any practice trials. **Figure 1** shows a trial event structure for the experimental paradigm.

### 2.3 Participants

39 patients [20 identifying as female, 19 as male; mean age 37.8 years (± 13.3)] diagnosed with major depressive disorder and 42 healthy control subjects [22 identifying as female, 20 as male; mean age 38.0 years (± 13.3)] were included into this study (for descriptives see **Table 1**). Gender distribution (X^2^(1,81)=0.01, p=0.92) and age (Student’s t(79)=-1.04, p=0.3) did not differ significantly between groups.

**Table 1:** Group descriptives.

	Diagnosis	Age
Mean	MDD	37.8
	Control	38.0
Median	MDD	35.0
	Control	32.5
Standard deviation	MDD	13.1
	Control	13.1
Minimum	MDD	19.0
	Control	18.0
Maximum	MDD	61.0
	Control	59.0

Patients were in-patients at the Department of Psychiatry at the University Hospital of Cologne, Germany. All patients had been admitted to a psychotherapy focused ward after diagnosis of major depressive disorder according to ICD-10 (43). Patients were only included after confirmation of the diagnosis according to DSM5 based on their patient file (44). Major depressive disorder according to ICD-10 had to be the current diagnosis for treatment at the time of inclusion. Patients were not included if they had been diagnosed with any comorbid psychiatric or neurological disorder, in particular bipolar disorder, schizoaffective disorder, and personality disorders. Patients were not included if they were taking psychoactive drugs other than prescribed for treatment of major depressive disorder. Patients were not included if they were currently taking benzodiazepines regularly or had taken benzodiazepines within 72 hours prior to the experiment.

Control subjects were recruited from the community and included if they had no history of psychiatric or neurological disorder and if they were not regularly taking any psychoactive or illegal drugs.

After instructions and providing informed consent, all participants first completed the questionnaires, starting with the BDI and ending with the SoAS. They then proceeded to perform the temporal binding experiment. After completion, participants were debriefed and received monetary compensation of 10 Euro per hour.

All procedures were performed at the Department of Psychiatry at the University Hospital of Cologne, Germany in accordance with the ethical standards of the institutional and/or national research committee and with the 1964 Helsinki Declaration and its later amendments or comparable ethical standards. The study was approved by the Ethics Committee of the Medical Faculty of the University of Cologne (No. 17-349). Written informed consent was obtained from all individual participants included in the study.

### 2.4 Statistical analysis and predictions

We predicted that the major depressive disorder group would exhibit higher BDI scores and lower GSE scores than the control group (CG) and a negative correlation between these two scores. For between group differences, we conducted serial independent sample Mann-Whitney U tests across both groups.

For the SoAS, we developed two competing predictions. Either, scores would differ between MDD and CG, indicating a divergence of (explicit) Sense of Agency between both groups possibly reflecting the loss of control experience in patients with major depressive disorder; or they would not differ between both groups, indicating an unaltered (explicit) Sense of Agency in major depressive disorder. SoAS scores could either be negatively associated with BDI scores, and positively correlating with GSE scores; or be unrelated to either score. The former assumption would represent a conceptual overlap between the Sense of Agency and self-efficacy as well as provide evidence for Sense of Agency alterations corresponding to depressive symptoms. The latter would indicate a lack of evidence for any such a relationship.

To investigate correlations of interest, we calculated separate linear regression models for GSE and SoAS in relation to BDI scores.

Concerning the experimental measurements in the temporal binding paradigm, we planned to first analyze the CG group to evaluate the feasibility of our paradigm, i.e., showing that the current paradigm reliably induced temporal binding in healthy/control participants. For the CG group, we expected significant effects for the overall estimation of different interval durations reflecting the participants’ ability to effectively differentiate between longer and shorter durations (effect for INTERVALL). We also expected significant effects for AGENCY. Particularly, we expected a relative underestimation of operant intervals, which we would interpret as the temporal binding effect. Lastly, effects for the different stimuli were expected, as differences in time perception and temporal binding for auditory or visual stimulation have been reported before (41, 45, 46). In line with the last assumption, we expected significant interactions between the factors AGENCY and STIMULUS. We analyzed effects of experimental manipulations on time estimates by using a linear mixed effects model as recommended for repeated measures designs (47) with random effects for participants.

For methodological reasons, we did not perform outlier checks or outlier removals. Temporal binding is a time estimation bias (Engbert et al., 2008) between actions and subsequent events and is sometimes referred to as a “temporal magnitude estimation” (48, 49). Typically, temporal binding paradigms do not involve practice or memorization trials during which participants learn specific durations. Tasks primarily directed at time perception usually require memorized durations as reference intervals from which individuals or groups digress during different experimental manipulations. Notably, our paradigm lacked such a controlled reference interval. As a result, participants’ results are prone to anchoring effects (34, 35) which make it difficult to compare estimates to real judgements, and to investigate differences in interindividual differences in time perception.

Taking into account these inter individual response strategies we believe removing outliers would distort results by reinforcing an anchoring bias on the visual analogue scale. To account for inter individual differences and anchoring effects we counterbalanced and randomised conditions across and within blocks to partially control for participants response strategies. Additionally, mixed effects models have demonstrated considerable robustness to outliers, violations of distributional assumptions, and interindividual variance (e.g., 50, 51). We additionally report results for an analysis of the data using Z-Scores for time estimates (see **Supplementary Table S1**).

After analysis of the CG group, we planned focused group analyses of those conditions that revealed robust temporal binding effects in the CG group. Concerning (potential) group differences in temporal binding, we speculated that a significant effect of group affiliation (DIAGNOSIS) on the factor AGENCY would reflect alterations in implicit Sense of Agency. Potential interactions with other factors were investigated exploratorily, as the experimental temporal binding paradigm had previously not been used for major depressive disorder groups.

To investigate the relationship between SoAS as an explicit Sense of Agency measurement and temporal binding as an implicit Sense of Agency measurement, we further defined temporal binding as the difference between individual operant and observant conditions. As both temporal binding and the SoAS are considered to be associated with Sense of Agency, a relationship between the two variables was expected (42). However, there exist several reports indicating that explicit Sense of Agency and temporal binding, i.e., implicit Sense of Agency, do not necessarily correlate in healthy participants (31, 32, 52–55). Hence, we considered both outcomes plausible.

All analyses were performed using SPSS 25 (56) and the R based (57) software jamovi (58).

## 3 Results

### 3.1 Questionnaire scores and comparisons

Scores obtained from questionnaires are shown in **Table 1**. We calculated total scores for the BDI-II and the GSE. With the SoAS we assessed positive Sense of Agency and negative Sense of Agency. We calculated a total SoAS score by adding the results from the positive scale and the inverted results from the negative scale. To examine the assumed relationships between the different scores of the groups, we performed serial independent sample Mann-Whitney U tests between both groups. As expected, all three scores differed significantly between groups (p<0.001) (for full results see **Table 2**).

**Table 2:** Questionnaire descriptives and comparisons.

Group Descriptives						
	Group	N	Mean	Median	SD	SE
BDI	CG	41	3.80	4.00	2.58	0.403
	MDD	39	24.4	21.0	11.23	1.798
GSE	CG	41	32.05	33.00	3.93	0.614
	MDD	39	20.2	21.0	6.01	0.963
SoAS	CG	41	79.27	81.00	8.18	1.277
	MDD	39	67.0	71.0	13.20	2.113

**Table:** 

Independent Samples T-Test									
		Statistic	p	Mean difference	SE difference	95% Confidence Interval	Upper		Effect Size
						Lower			
BDI	Mann-Whitney U	40.0	<.001	-18.0		-23.00	-15.0	Rank biserial correlation	0.950
GSE	Mann-Whitney U	96.0	<.001	12.0		10.00	14.0	Rank biserial correlation	0.880
SoAS	Mann-Whitney U	336.5	<.001	11.0		7.00	17	Rank biserial correlation	0.579

We estimated correlations with BDI between groups and inter-questionnaire scores by calculating separate linear regression models for GSE and SoAS in relation to BDI scores.

As expected, we found a strong relationship between GSE and BDI scores (p<.001, R^2^=.650). If calculated for both groups separately, the regression model also reached statistical significance for the CG participants (p=.022, R^2^=.127) and for patients with major depressive disorder (p<.001, R^2^=.331).

**Figure 2:**
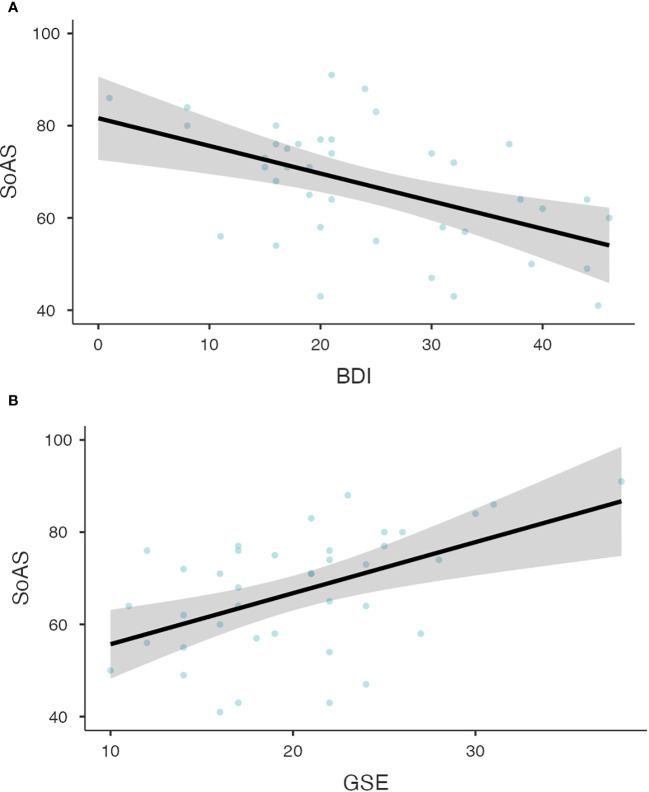
**(A)** Relationship between BDI and SoAS scores for patients with major depressive disorder. The Linear Regression Model for the relationship between BDI and SoAS scores shows the expected significant relationship between both variables (p<.001, R^2^=.260). Observed scores are depicted as grey dots for CG participants and black dots for patients. Panel **(B)** Relationship between GSE and SoAS scores for patients with major depressive disorder. The Linear Regression Model for the relationship between GSE and SoAS scores shows the expected significant relationship between both variables (p=.001, R^2^=.254). Observed scores are depicted as grey dots for CG participants and black dots for patients.

Linear regression models across both groups revealed a strong relationship between both BDI and SoAS (p<.001, R^2^=.405, **Figure 2A**), as well as between GSE and SoAS (p<.001, R^2^=.461, **Figure 2B**). The regression models again reached statistical significance when calculated for both groups separately with SoAS and BDI (CG: p=.032, R^2^=.113; MDD: p<.001, R^2^=.260) as well as SoAS and GSE (CG: p<.001, R^2^=.379; MDD: p=.001, R^2^=.254). **Figure 2** shows results for testing our hypotheses of a relationship between SoAS and BDI as well as SoAS and GSE for the patient group.

The overall results from comparisons with the SoAS scores did not seem to be particularly driven by either negative Sense of Agency nor positive Sense of Agency with a slight advantage for positive Sense of Agency. The results from the comparisons can be found in the supplement (**Supplementary Table S2**).

### 3.2 Experimental results

Effects of experimental manipulations on time estimates were analyzed using a linear mixed effects model as recommended for repeated measures designs (47) with random effects for participants. Visual inspection of residual plots did not suggest deviations from homoscedasticity or normality. Prior to analysis participants were individually screened for random response behavior. All participants showed a linear trend between the three intervals on visual inspection, reflecting their overall engagement in the task and their ability to differentiate intervals.

#### 3.2.1 Analysis of the CG group: validating the current temporal binding paradigm

As stated in the methods, a complex temporal binding paradigm was applied to account for potential alterations in implicit Sense of Agency across different stimulus modalities and time intervals. Therefore, we first had to ensure that the current paradigm resulted in robust temporal binding effects in the CG. Thereafter, we would compare the performances of both groups (CG, MDD) in those conditions of the current paradigm that revealed significant temporal binding effects for the control participants.

**Figure 3:**
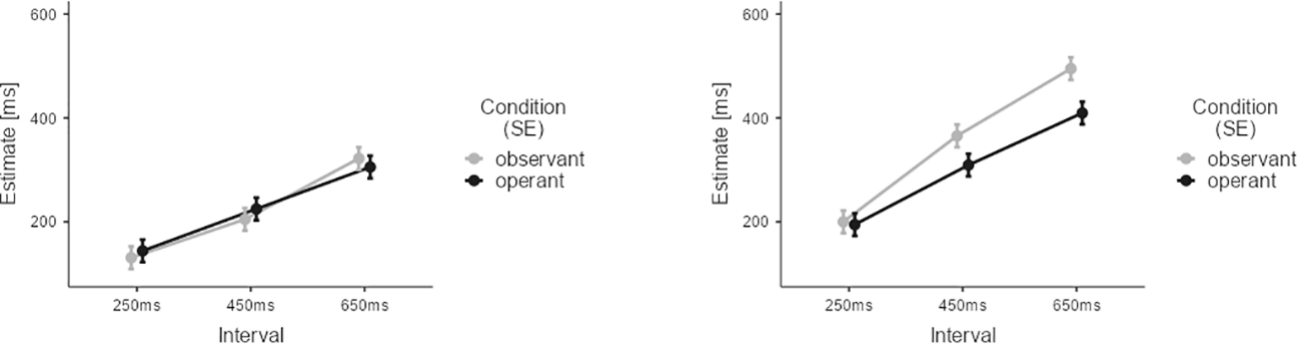
Results of the temporal binding experiment for the CG group. Estimates from observant trials are depicted in grey. Estimates from operant trials are depicted in black. Lower estimates for operant trials indicate temporal binding. Error Bars indicate Standard Error (SE).

For the within group analysis of the CG, we adopted a three-factorial model with time estimates as the dependent variable. The three factors were AGENCY (operant vs. observant), STIMULUS (visual vs. auditory), and INTERVAL (250ms, 450ms, 650ms). Results for experimental effects are depicted in **Figure 3**. Results after Z-scoring did not differ from non-standardised results and are reported in the supplement.

For CG participants, all three main effects reached statistical significance: AGENCY (M=-21.8, SE=4.66, t=-4.67, p<0.001), STIMULUS (M=-107.0, SE=4.66, t=22.97, p<0.001), and INTERVAL (M_450ms-250ms_=108.7, SE=5.70, t=19.06, p<0.001; M_650ms-250ms_=215.6, SE=5.70, t=37.80, p<0.001). The following interactions between factors also reached significance: AGENCY and STIMULUS (M=-54.5, SE=9.32, t=-5.86, p<0.001), AGENCY and INTERVAL (M_450ms-250ms_=-22.0, SE=11.41, t=1.93, p=0.05; M_650ms-250ms_=-54.8, SE=11.41, t=-4.81, p<0.001), and STIMULUS and INTERVAL (M_450ms-250ms_=62.7, SE=11.41, t=5.50, p<0.001; M_650ms-250ms_=78.6, SE=11.41, t=6.89, p<0.001). The three-way interaction was also statistically significant (M_450ms-250ms_=-57.9, SE=22.82, t=-2.54, p=0.011; M_650ms-250ms_=-50.4, SE=22.82, t=-2.21, p=0.022). These results confirmed our initial hypotheses concerning temporal binding in the CG group (see **Supplementary Tables S1.1** and **S1.2** for results after Z-scoring). Data analysis revealed strong temporal binding for the auditory condition, increasing with interval duration. Yet, no significant temporal binding was detectable for visual stimulation. The current findings in healthy persons are in line with the existing literature (41, 42, 45), as we will discuss in more detail in the discussion section. Consequently, we explored any potential group differences for the auditory conditions and visual conditions separately, since only the former elicited robust temporal binding effects in the CG.

#### 3.2.2 Temporal binding in major depressive disorder - analysis of visual stimulus conditions across both groups

**Figure 4:**
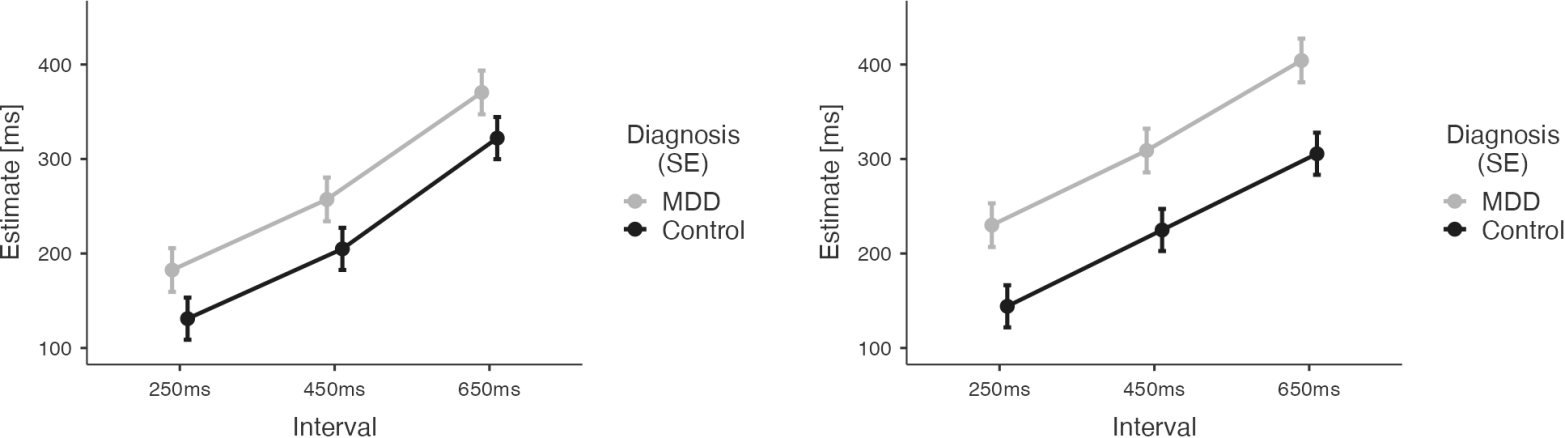
Results of the Temporal Binding Experiment with visual stimuli across the two groups (MDD, CG). Estimates by the patient group are depicted in grey. Estimates by the CG group are depicted in black. Error Bars indicate Standard Error (SE). Results show larger estimates of tone-flash-intervals (observant, left) and action-flash intervals (operant, right) for patients with major depressive disorder.

We applied a mixed effects model across data from both groups for the visual conditions only. The differential group results of this model are depicted in **Figure 4**.

Analysis revealed overestimation of durations for the patient group (main effect of DIAGNOSIS: M= -70.14, SE= 30.42, t -2.306, p= 0.024). The significant effect of the factor INTERVAL (M_450ms-250ms_=77.09, SE=5.67, t=13.59, p<0.001; M_650ms-250ms_=178.690, SE=5.67, t=31.50, p<0.001) indicated both groups’ ability to differentiate durations. There was also a main effect of AGENCY (M= 24.92, SE=4.63, t= 5.38, p<0.001) reflecting an influence of action on time estimates for visual stimuli. However, the direction of the AGENCY effect was opposite than expected: there was an increase of time estimates for operant conditions compared to observant conditions (see **Supplementary Table S1.3** for results after Z-scoring).

This unexpected finding was driven by an increase of time estimates in the patient group for the operant visual conditions (compared to the observant visual conditions), while the time estimates in these conditions did not differ for CG participants, resulting in a significant interaction between DIAGNOSIS and AGENCY (M=-38.82, SE= 9.26, t=-4.19, p<0.001).

No other interactions reached statistical significance.

#### 3.2.3 Temporal binding in major depressive disorder - analysis of auditory stimulus conditions across both groups

**Figure 5:**
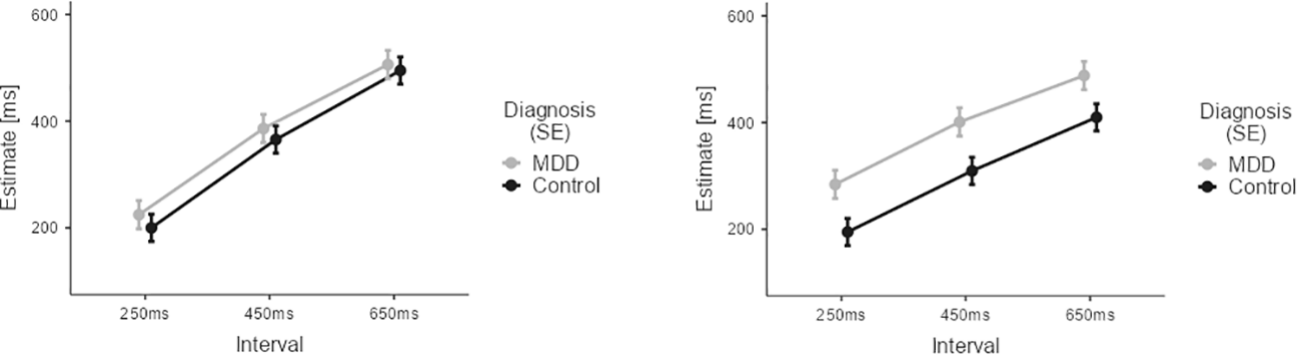
Results of the Temporal Binding Experiment with auditory stimuli across the two groups (MDD, CG). Estimates by the patient group are depicted in grey. Estimates by the CG group are depicted in black. Error Bars indicate Standard Error (SE). Results suggest that the interaction effect of agency (observant vs. operant) and diagnosis on time estimates is primarily driven by differences in duration perception during operant trials involving intentional action.

We applied a mixed effects model across data from both groups for the auditory conditions only. The differential group results of this model are depicted in **Figure 5**.

In contrast to the visual conditions, the two groups (CG, MDD) did not significantly differ in overall estimation ability for auditory stimuli: non-significant main effect DIAGNOSIS (M=-52.62, SE=34.86, t=-1.509, p=0.135).

Again, a significant effect of the factor INTERVAL (M_450ms-250ms_=139.68, SE=6.33, t=22.054, p<0.001; M_650ms-250ms_=242.828, SE=6.33, t=39.3, p<0.001) indicated that overall both groups were able to correctly differentiate the different time intervals in the auditory conditions.

Across groups, there was also a main effect of AGENCY (M=-15.23, SE=5.17, t=-2.95, p=0.003) reflecting the overall presence of temporal binding in the auditory conditions.

Of note, a significant interaction between AGENCY and INTERVAL (M_450ms-250ms_=-47.74, SE=12.67, t=-3.77, p<0.001; M_650ms-250ms_=-78.73, SE=12.67, t=-6.22, p<0.001) reflected that temporal binding was influenced by interval duration.

Importantly, we found a highly significant interaction between the factors DIAGNOSIS and AGENCY (M=-67.60, SE=10.34, t=-6.54, p<0.001). This interaction confirmed that temporal binding was present in the CG group, but temporal binding was diminished in the patient group for the auditory conditions.

No other interactions reached statistical significance (see **Supplementary Table S1.4** for results after Z-scoring).

#### 3.2.4 Analysis of temporal binding and SoA

For analyzing the individual measures of implicit Sense of Agency and explicit Sense of Agency (as well as implicit Sense of Agency and GES), implicit Sense of Agency was operationalized as the individual/participant-wise mean difference between the time estimations of the observant and operant conditions in the constellation with the most pronounced temporal binding effects (i.e., 650ms interval conditions with auditory stimuli). Scores above zero indicated temporal binding.

Then, we investigated the potential association between implicit Sense of Agency measures (derived from the temporal binding paradigm) and explicit Sense of Agency scores (derived from the SoAS) and with GSE scores. A general linear model of the Sense of Agency measures with SoAS scores did not yield any statistically significant correlation, neither for the entire data set (F(1,80)=0.69, p=0.410, η²p=0.01), nor for the CG participants (F(1,41)=0.04, p=0.85, η²p=0.001), nor for the patient group (F(1,38)=0.008, p=0.931, η²p<0.0001).

## 4 Discussion

This study combined questionnaire-based measurements with an experimental paradigm for temporal binding. All assessed variables targeted the experience of loss of control in major depressive disorder. Thereby, we aimed at elucidating the relationship between Sense of Agency, self-efficacy, and the severity of depressive symptoms in major depressive disorder.

As expected, BDI and GSE scores for depressive symptoms and self-efficacy correlated strongly and differed significantly between groups. SoAS scores for sense of agency also differed significantly between groups. Furthermore, the scores for explicit Sense of Agency correlated significantly both with BDI and GSE. Although self-efficacy and Sense of Agency describe distinct phenomena (6), both are linked to an experience of control over the external world and one’s own life. Depending on context, both terms might even mean similar aspects and share a common pathway (4, 5, 7). Our finding suggests a conceptual and psychopathological connection between Sense of Agency and self-efficacy. Alternatively, it can be interpreted as related yet independent decreases of Sense of Agency and self-efficacy in major depressive disorder. Due to the apparent differences in item content in both scales and the previously reported lack of correlation between other measures of self-efficacy and the SoAS (37), the latter interpretation appears more likely. The correlation of GSE and SoAS scores in major depressive disorder motivates further investigation of the connection between the two concepts. In either case, the results suggest a profound loss of the experience of being in control for patients with major depressive disorder. It is noteworthy that symptom severity as measured with the BDI-II ranged from very low to high scores for the patient group. This inclusion of participants with lower scores on the BDI may bias the regression statistics. We therefore suggest a replication of this investigation with a larger sample size or with a selection of patients based on symptom severity.

As stated above, changes in (explicit) Sense of Agency are usually not discussed as a common feature to major depressive disorder. Our findings of reduced SoAS scores correlating with symptom severity indicate that the experience of loss of control in major depressive disorder might be even more pronounced and generalized than assumed. Conceptually, this would entail not only negative changes in self-efficacy expectations but further pertain to control over bodily movements and functions.

Consistent with previous studies, the current, complex paradigm elicited robust temporal binding effects in the control group for auditory, but not for visual stimuli (41, 42, 45). Moreover, in the control group temporal binding was pronounced for longer intervals involving auditory stimuli. Thus, we replicated previous findings concerning differential effects of stimulus modality and interval duration on temporal binding (41, 45, 46).

Our experimental results show a distinct difference in temporal binding for auditory stimuli between healthy participants and participants diagnosed with major depressive disorder. This alteration was driven by a lack of relative underestimation of time intervals when participants pressed a button during operant conditions, as compared to estimating a duration between two auditory stimuli not involving an intentional action, as no group differences were observed for the observant conditions (see **Figure 5**). Considering the findings of an interrelated decrease in self-efficacy and in Sense of Agency, this decrease of a temporal binding for motor actions may further hint at a diminished action control in major depressive disorder involving both explicit and implicit mechanisms. Our findings suggest that a loss of control over physical actions is broadly altered in major depressive disorder. As we did not find any correlation between temporal binding and SoAS, our results provide evidence that implicit and explicit Sense of Agency measures do not necessarily correlate systematically, but may depend on additional factors (52–54).

Despite this lack of a correlation between the implicit and the explicit Sense of Agency measures, our finding concerning the difference in temporal binding between the two groups may shed further light on loss of control experiences in major depressive disorder. Particularly for auditory conditions, temporal binding was present for the CG participants. Yet, in the auditory conditions temporal binding levelled out for the patient group. This disappearance of temporal binding in major depressive disorder appears to be driven by a change in time perception for those intervals following intentional action in the context of auditory stimuli (see **Figure 5**).

Recent discussions suggest a link between temporal binding and multisensory processing (23, 26). The underlying research implicates, that temporal binding poses a special case of causal inference (22, 25). Related events are processed simultaneously across an extended temporal binding window when causally related (Jagini, 2012). The simultaneity of neural processing underlies the illusion of the two perceptual events being closer together. In temporal binding paradigms, the binding of an event to a causal motor action appears to be stronger for auditory as compared to visual events (41, 45, 46).

This is in line with recent reports of temporal binding as a procedural confound dependant on temporal grouping mechanisms (59, 60) instead of being related to Sense of Agency. The perceptual shift between two events is thus not explained by intentionality and agency, but by a perceptual shift grouping two subsequent events together and hence increasing their perception as belonging together (59).

Evidence has been produced that the brain processes auditory information differently in patients with major depressive disorder. For instance, in an EEG-study Kähkönen and colleagues (61) found that early auditory processing in an odd-ball paradigm was impaired in patients. In two fMRI studies (62, 63) the authors demonstrated that sine tone presentation resulted in distinct brain activation between patients and controls, both in acute and remitted states.

Our paradigm suggests that these findings may extend to multisensory processing involving motor and audio stimuli. In our results, a group difference in time estimates was detected only for conditions involving motor action. This may suggest, that patients with major depressive disorder may process proprioceptive and other body related information differently from control participants. We suggest that future studies should combine electrophysiological or functional measurements with a temporal binding task involving different sensory modalities to substantiate our hypothesis of altered motor-audio-processing in major depressive disorder.

Although our results are interpretable as a change in implicit Sense of Agency for motor-audio sequences in major depressive disorder, the overall similarity of auditory and visual trials combined with the usual assumption of a general feeling of loss of control in major depressive disorder irrespective of stimulus quality makes a change in time perception for action-event processing involving auditory stimuli more likely (59).

Yet, for visual stimuli our analysis revealed in patients a significant general overestimation of intervals. During visual trials, perceptual information was more complex as compared to during auditory trials. During passive, auditory trials, stimulation contained a single stimulus modality, i.e., auditory stimulation. During all other trials, perceptual input was multisensory with a combination of either auditory and visual (passive/visual trials) or proprioceptive (active) and visual/auditory information. We propose that increasing complexity in multisensory stimulus integration led to the longer interval estimates of patients in the visual conditions of the current paradigm.

The study design, however, is subject to limitation. We designed the experimental paradigm in accordance with earlier studies (41, 42) to provide a comprehensive experimental approach to major depressive disorder as a yet understudied group. The resulting complexity of the design, particularly the involvement of two stimulus modalities, on the one hand resulted in the detection of a (specific) differential temporal binding effect for auditory stimuli. On the other hand, randomization of stimulus presentation during blocks might have increased complexity for perceptual processing. In other words, participants might have been confused by the random changes between stimulus modalities. It is plausible that this in turn may have caused more subtle binding effects (e.g., for visual stimulation and lower durations) to be less detectable. The patient group may have been more susceptible to this negative influence of paradigm complexity, which in turn might explain differences in temporal binding between the groups. This is of particularly interest as our study is potentially underpowered to detect more subtle differences, particularly between groups. For example, to detect medium effect sizes (d = 0.50) power is as low as 60% given the current sample size.

As we did not fing a direct correlation between results from the temporal binding task and the SoAS we cannot assume temporal binding results as a decreased implicit Sense of Agency. Accordingly, we assume our experimental results to indicate specific difference in perception during multisensory duration estimation tasks. This again is in line with earlier findings concerning the underlying mechanisms of temporal binding which have challenged the effect as an implicit correlate to Sense of Agency (23, 24, 59).

We used the SoAS as a trait measure for agency. Although our primary target were trait alterations during major depressive disorder, it is important to note that experienced agency is highly situational. Although these situational states may directly be influenced by trait variables, the SoAS does not do full justice to potential changes in Sense of Agency. Particularly considering the difference in temporal binding between the two groups, it is of interest whether state dependant trial by trail differences in Sense of Agency, as for example assessed by trial by trial queries of an experience of agency, may also be different between groups and correlate with the difference in temporal binding.

This is especially interesting concerning conceptual distinctions between self-efficacy and Sense of Agency. While the former denotes the expectation of being able to reach a desired action outcome, the latter relates to the ongoing sense of being and having been in control. The two scales used herein assess the overall capacity for both phenomena. Yet, with both being situationally dependant, trial by trail assessments with outcome manipulations may yield results on their dependability on and/or correlation with multisensory processing as measured with temporal binding. Furthermore, the SoAS distinguished between positive and negative Sense of Agency, which may correlate with other measures, including self-efficacy (5). Future studies involving larger samples should direct further attention at these potential connection.

Considering these limitations, our study may inspire a new research direction for major depressive disorder. Future temporal binding and time estimation paradigms involving patients with major depressive disorder might benefit from reduced perceptual and predictive complexity and yield more concise results across perceptual modalities.

The SoAS was neither explicitly designed to assess symptoms of major depressive disorder, nor has a German translation been sufficiently validated. The explicit Sense of Agency deficit in our patient group may be secondary to a negative bias in the patient group (64) or have been influenced by the fixed order in which questionnaires were administered. We argue that our results call for a larger scale application of the SoAS both across clinical and across cultural populations.

Overall, we suggest that the relationship between the experience of Sense of Agency and self-efficacy and their respective alterations in major depressive disorder may broaden our understanding of the disorder and its key symptoms. The results suggest a profound experience of loss of control in severe major depressive disorder going beyond what the concept of self-efficacy may address. An integration of Sense of Agency alterations into current research on the experience of control will substantially improve our understanding of patient´s first person experiences.

Importantly, changes in control related phenomena in major depressive disorder not only involve experiential states as measured by questionnaires. They also affected temporal binding as an implicit Sense of Agency measure. Future research integrating both explicit psychopathological descriptives with implicit behavioral measures holds the potential of fostering a better understanding of depressive states as well as opening new avenues for diagnosis and patient evaluation.

## Data availability statement

The raw data supporting the conclusions of this article will be made available by the authors, without undue reservation.

## Ethics statement

The studies involving humans were approved by Ethics Committee of the Medical Faculty of the University of Cologne. The studies were conducted in accordance with the local legislation and institutional requirements. The participants provided their written informed consent to participate in this study.

## Author contributions

DV: Conceptualization, Data curation, Formal analysis, Investigation, Methodology, Project administration, Visualization, Writing – original draft, Writing – review & editing. MJ: Conceptualization, Investigation, Writing – review & editing. PW: Conceptualization, Investigation, Supervision, Writing – review & editing. KV: Conceptualization, Investigation, Supervision, Writing – review & editing.
