# Supplementary material for: Temporal binding and sense of agency in major depression
Source: Front Psychiatry. 2024 Apr 5;15:1288674. doi: 10.3389/fpsyt.2024.1288674 (PMC11027068; doi:10.3389/fpsyt.2024.1288674)
Supplement: Supplementary file 1 [file DataSheet_1.pdf]

Supplement

Tables S1: Results after Z-scoring time estimates. Z-Scoring did not change the statistical results.

Table S1.1: Results across both groups after Z-scoring.

	F	Num df	Den df	p
Agency	1.848	1	9616.0	0.174
Stimulus	759.781	1	9616.0	<.001
Interval	1199.380	2	9616.0	<.001
Diagnosis	3.849	1	79.0	0.053
Agency ✻ Stimulus	31.735	1	9616.0	<.001
Agency ✻ Interval	16.662	2	9616.0	<.001
Stimulus ✻ Interval	38.901	2	9616.0	<.001
Agency ✻ Diagnosis	55.724	1	9616.0	<.001
Stimulus ✻ Diagnosis	6.045	1	9616.0	0.014
Interval ✻ Diagnosis	0.102	2	9616.0	0.903
Agency ✻ Stimulus ✻ Interval	6.693	2	9616.0	0.001
Agency ✻ Stimulus ✻ Diagnosis	4.075	1	9616.0	0.044
Agency ✻ Interval ✻ Diagnosis	0.159	2	9616.0	0.853
Stimulus ✻ Interval ✻ Diagnosis	0.622	2	9616.0	0.537
Agency ✻ Stimulus ✻ Interval ✻ Diagnosis	0.210	2	9616.0	0.810

Note. Satterthwaite method for degrees of freedom

Table S1.2: Results for the CG group after Z-scoring.

	F	Num df	Den df	p
Agency	21.85	1	4987	<.001
Stimulus	527.62	1	4987	<.001
Interval	714.28	2	4987	<.001
Agency ✻ Stimulus	34.28	1	4987	<.001
Agency ✻ Interval	11.70	2	4987	<.001
Stimulus ✻ Interval	26.57	2	4987	<.001
Agency ✻ Stimulus ✻ Interval	3.81	2	4987	0.022

Note. Satterthwaite method for degrees of freedom

Table S1.3: Results for comparisons of visual stimuli between groups after Z-scoring.

	F	Num df	Den df	p
Agency	28.947	1	4769.0	<.001
Interval	499.269	2	4769.0	<.001
Diagnosis	5.318	1	79.0	0.024
Agency ✻ Interval	3.209	2	4769.0	0.040
Agency ✻ Diagnosis	17.563	1	4769.0	<.001
Interval ✻ Diagnosis	0.133	2	4769.0	0.875
Agency ✻ Interval ✻ Diagnosis	0.397	2	4769.0	0.673

Note. Satterthwaite method for degrees of freedom

Table S1.4: Results for comparisons of auditory stimuli between groups after Z-scoring.

	F	Num df	Den df	p
Agency	8.6752	1	4768.0	0.003
Interval	775.8995	2	4768.0	<.001
Diagnosis	2.2780	1	79.0	0.135
Agency ✻ Interval	19.6142	2	4768.0	<.001
Agency ✻ Diagnosis	42.7314	1	4768.0	<.001
Interval ✻ Diagnosis	0.5810	2	4768.0	0.559
Agency ✻ Interval ✻ Diagnosis	0.0331	2	4768.0	0.967

Note. Satterthwaite method for degrees of freedom

Tables S2: Results for comparisons between questionnaire scores and SoAS sub scales (positive and negative SoA)

Tables S2.1: Results from linear regression model between positive SoA and BDI.

Linear Regression

Model Fit Measures

Model	R	R^2
1	0.535	0.286

Model Coefficients - SoAS-positive

Predictor	Estimate	SE	t	p
Intercept	35.120	0.9586	36.64	<.001
BDI	–0.282	0.0505	–5.59	<.001

Tables S2.2: Results from linear regression model between negative SoA and BDI.

Linear Regression

Model Fit Measures

Model	R	R^2
1	0.515	0.265

Model Coefficients - SoAS-negative

Predictor	Estimate	SE	t	p
Intercept	11.231	1.0102	11.12	<.001
BDI	0.282	0.0532	5.31	<.001

Tables S2.3: Results from linear regression model between positive SoA and GSE.

Linear Regression

Model Fit Measures

Model	R	R^2
1	0.652	0.425

Model Coefficients - SoAS-positive

Predictor	Estimate	SE	t	p
Intercept	16.035	2.0849	7.69	<.001
GSE	0.578	0.0761	7.59	<.001

Tables S2.4: Results from linear regression model between negative SoA and GSE.

Linear Regression

Model Fit Measures

Model	R	R^2
1	0.546	0.298

Model Coefficients - SoAS-negative

Predictor	Estimate	SE	t	p
Intercept	28.346	2.3915	11.85	<.001
GSE	−0.503	0.0873	−5.76	<.001

Tables S2.5: Results from linear regression model between positive SoA and BDI for the MDD group.

Linear Regression

Model Fit Measures

Model	R	R^2
1	0.372	0.139

Model Coefficients - SoAS-positive

Predictor	Estimate	SE	t	p
Intercept	33.876	2.5868	13.10	<.001
BDI	–0.236	0.0966	–2.44	0.020

Tables S2.6: Results from linear regression model between negative SoA and BDI for the MDD group.

Linear Regression

Model Fit Measures

Model	R	R^2
1	0.340	0.115

Model Coefficients - SoAS-negative

Predictor	Estimate	SE	t	p
Intercept	12.598	2.856	4.41	<.001
BDI	0.234	0.107	2.20	0.034

Tables S2.7: Results from linear regression model between positive SoA and GSE for the MDD group.

Linear Regression

Model Fit Measures

Model	R	R^2
1	0.572	0.328

>

Model Coefficients - SoAS-positive

Predictor	Estimate	SE	t	p
Intercept	14.456	3.356	4.31	<.001
GSE	0.677	0.159	4.25	<.001

Tables S2.7: Results from linear regression model between negative SoA and GSE for the MDD group.

Linear Regression

Model Fit Measures

Model	R	R^2
1	0.331	0.110

Model Coefficients - SoAS-negative

Predictor	Estimate	SE	t	p
Intercept	26.930	4.206	6.40	<.001
GSE	−0.427	0.200	−2.14	0.039
